# Free Gingival Graft as a Single Step Procedure for Treatment of Mandibular Miller Class I and II Recession Defects 

**DOI:** 10.29252/wjps.8.1.12.

**Published:** 2019-01

**Authors:** Lata Goyal, Narender Dev Gupta, Namita Gupta, Kirti Chawla

**Affiliations:** 1 Department of Dentistry, All India Institute of Medical Sciences, Rishikesh, India;; 2 Department of Periodontics and Community Dentistry, Dr. Ziauddin Ahmad Dental College, Aligarh Muslim University, Aligarh India;; 3 Department of Periodontology, Jamia Millia Islamia, New Delhi, India

**Keywords:** Perioplastic surgery, Aethetics, Gingival recession

## Abstract

**BACKGROUND:**

Gingival recession is a frequent issue encountered by both the clinician and the patient. This study was aimed to assess the predictability of the free gingival graft as a single step procedure in terms of root coverage and aesthetics in Miller Class I and II mandibular gingival recession.

**METHODS:**

Ten patients (4 males, 6 females) aged 25-30 years with a total of 12 mandibular sites having Miller class I and II recession were selected. All recession sites were treated with single step free gingival graft procedure. Clinical parameters like recession depth, recession width, width of attached gingiva, probing depth and clinical attachment level were recorded at baseline, 6 and 9 months. Visual analog score at 1, 6 and 9 months postoperatively was provided.

**RESULTS:**

There was a reduction in mean recession depth from 3.66±1.20 to 0.91±0.99 mm suggesting coverage of 82% over a period of 9 months. There was statistically significant gain in clinical attachment level and width of attached gingiva. Aesthetically, it was acceptable by patients as measured by visual analog scores.

**CONCLUSION:**

Free gingival graft as a single step procedure is acceptable in terms of root coverage and aesthetics.

## INTRODUCTION

Patient’s growing interest in aesthetics has lead to refinement in the goals of mucogingival surgery. Gingival recession is a frequent issue encountered by both the clinician and the patient. It is defined as apical displacement of gingival margin from the cementoenamel junction.^1^ Main indication of root coverage includes aesthetics, root sensitivity, management of root caries and cervical abrasion.^2^ Dorfam stated that if marginal tissue can be maintained free of inflammation, treatment of recession should not be considered,^3^ but according to Miller, it is quite predictable and produces patient’s satisfaction.^4^ Various factors have to be taken care in selecting the procedure of choice for root coverage like extent of recession, width of attached gingiva, aesthetic concern, patient comfort, and the position of tooth in the arch.^5^^,^^6^

There are various surgical techniques available for root coverage like rotational flaps,^7^ coronally advanced flap,^8^ free gingival graft,^4^ guided tissue regeneration,^9^ connective tissue graft and combination of these.^10^ Despite of the advances in technique of correction of gingival recession, free gingival graft continues to be a reliable procedure for increasing the width of keratinized gingiva and stopping the progression of gingival recession.^11^ At present, even though the free gingival grafts have lost their race to subepithelial connective tissue grafts as far as root coverage is concerned, they still hold an edge in considerations like being simple, multiple teeth can be treated at one time, easy tissue handling, and can be performed when keratinized gingiva adjacent to involved is insufficient.^12^

Main disadvantage of free gingival graft is lack of predictability in terms of aesthetics. As most of the studies are done in Caucasian population this study was aimed to assess the predictability of free gingival graft in terms of root coverage and color match in mandibular recession defects obtained in a regional North Indian population. Mandibular teeth face more challenge and difficult to treat because of certain anatomical factors like thin gingival biotype, shallow vestibular depth and high frenum attachment.^13^ Keeping in mind these anatomical factors free gingival graft could be a procedure of choice in treatment of recession defects. Moreover, in Indian scenario, gingiva of the patient is characterized by high melanin pigmentation and better level of esthetics can be achieved by free gingival graft.^14^ In this study, free gingival graft as a single step procedure for treatment of mandibular miller class I and II recession defects has been assessed.

## MATERIALS AND METHODS

**Fig. 1:**
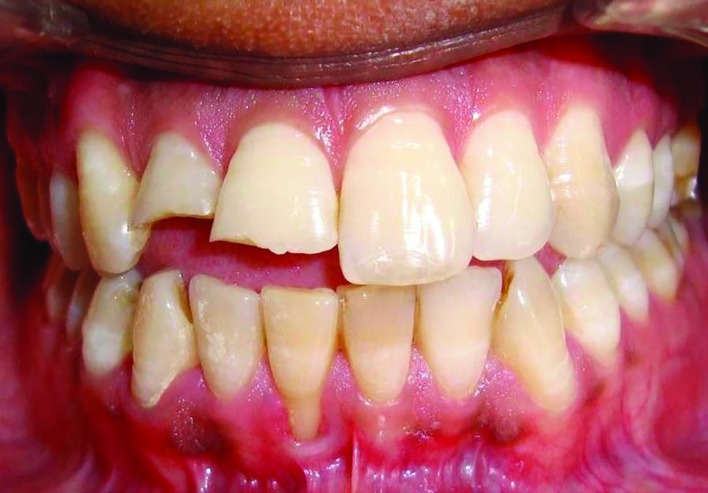
Preoperative view showing Miller Class II recession associated with tooth 41

**Fig. 2:**
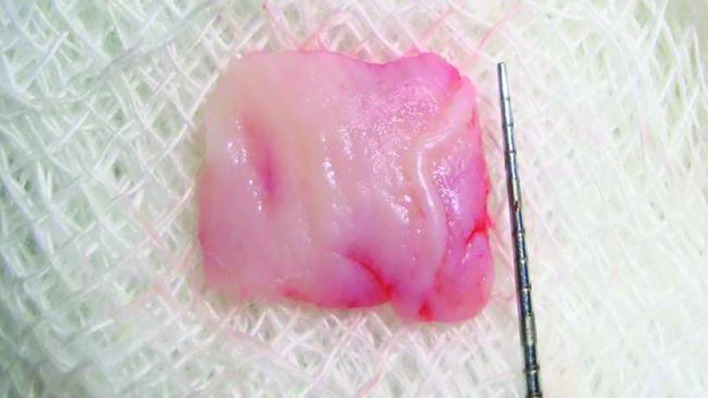
Free gingival graft

The study group consisted of 10 patients (4 males, 6 females) aged 25-30 years who were referred to the Department of Periodontics. A total of 12 mandibular sites were selected having Miller class I and II recession (Figure 1 and 2). Gingival biotype of teeth adjacent to gingival recession was assessed by transgingival probing method. Patient’s having thin gingival biotype (<1 mm) of teeth adjacent to recession were selected for the study. Tooth selected for root coverage were vital, non carious and without cervical abrasion. Initial therapy was consisted of scaling and root planing and oral hygiene was reinforced by giving oral hygiene instructions. After 2 months, patients’ periodontium was evaluated and selected sites without any signs of gingival inflammation and bleeding on probing were selected. 

Following parameters were evaluated at mid buccal aspect at baseline, 6 and 9 months using UNC 15 (University of North Calorina) probe by the same examiner to avoid any bias. (i) Recession depth (RD) was measured as distance from cementoenamel junction (CEJ) to the gingival margin. (ii) Recession width (RW) was measured as the distance across the buccal surface at the CEJ level. (iii) Probing depth (PD) was measured as the distance from the gin­gival margin to the base of the sulcus in millimeters. (iv) Clinical attachment level (CAL) was measured as the distance in millimeters from the cementoenamel junction to the base of the sulcus and assessed from recession depth and probing depth. (v) Width of attached gingiva (WAG) was measured as the distance between the mucogingival junction and the projection on the external gingival surface of the most apical portion of the gingival sulcus or the periodontal pocket.

**Fig. 3:**
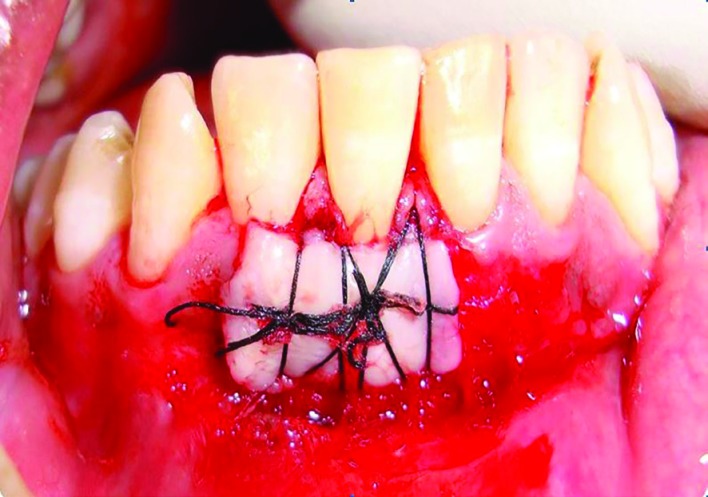
Graft is placed to prepared mucoperiosteal bed and sutured by interdental, horizontal and circumferential suture

A visual analog scale (VAS) was used to analyze the color match of the grafts. To determine the color match, the 0-10 scale criteria, in that 0=no color match, 10=absolute color match and <5=unsatisfactory. The scoring was done at the end of 1, 3 and 6 months in all the patients. After achieving adequate local anesthesia, exposed root surface was planed thoroughly. The horizontal incision was given extending from the line angle of adjacent teeth on either side of the recession at the level of CEJ. Two vertical incisions were made to extend well into the alveolar mucosa at the distal terminal of horizontal incision. A split thickness flap was elevated without disturbing periosteum. Root biomodification with citric acid was done for 5 min. The amount of donor tissue needed was accurately determined by using a foil template. The area between first and second premolar which had a greater thickness was selected to harvest the donor tissue (Figure 3). 

**Fig. 4:**
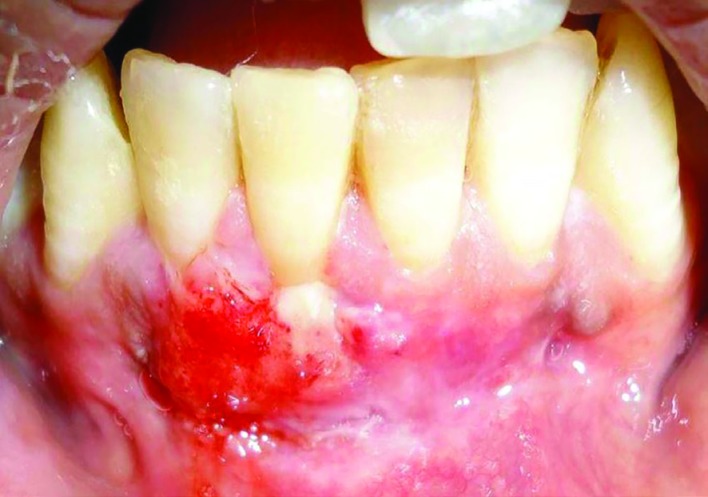
Surgical site after removal of sutures

**Fig. 5:**
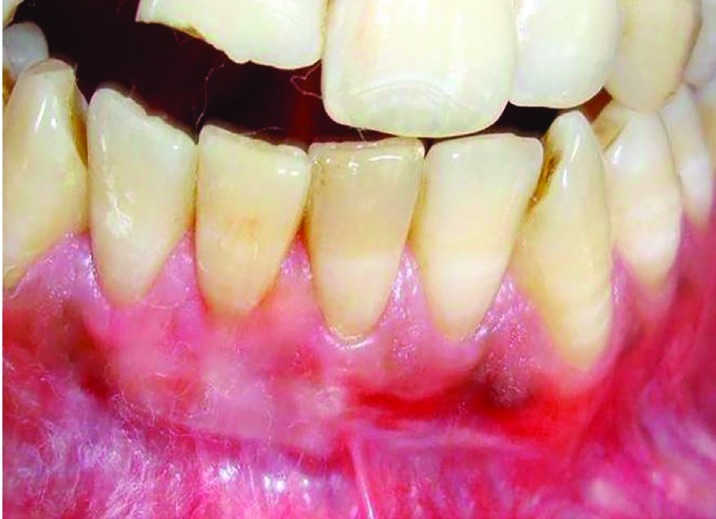
9 month postoperative view

The graft was placed on the recipient bed and suturing was done as described by Holbrook and Oschenbein^15^ (Figure 4). Periodontal dressing was placed at the surgical site. The subjects were asked to refrain from tooth brushing at the surgical site for two weeks. Totally, 0.12% chlorhexidine mouth rinsing twice daily for 3 weeks and a course of antibiotics including amoxicillin 500 mg thrice daily and 400 mg of ibuprofen thrice daily for 5 days. The pack was removed 2 weeks post operatively (Figure 5). Subjects were recalled at 3, 6 and 9 months for followup. Clinical Parameters were recorded at 6 and 9 months. There was uneventful healing without any complications.

## RESULTS

Results suggest the success of free gingival graft as a procedure for recession in terms of root coverage and esthetics in the studied population. At baseline, the mean recession depth was 3.66±1.20; which reduced to 0.91±0.99 mm at the end of 9 months suggesting coverage of 82% (Table 1). Mean root coverage after 9 months was 2.75±0.62 mm and 82.22±19.69%. Visual analogue scale was 3.83±0.93 at 1 month postoperative, 5.41±1.08 after 6 months, which met the satisfactory criteria post-op 9 months with a mean of 6±1.34 (*p*=0.0001).

**Table 1:** Mean clinical parameters

**Parameter**	**Baseline**	**9 months**	**p value**
RD (mm)	3.66±1.20	0.91±0.99	<0.0001*
RW (mm)	3.4±0.67	0.21±0.34	<0.0001*
CAL (mm)	5.75±1.60	2.08±0.98	<0.0001*
PD (mm)	2.08±0.66	1.25±0.45	<0.0001*
WAG (mm)	0.33±0.47	2.92±0.79	<0.0001*

* Stastically significant. RD=recession depth, RW=recession width, CAL=clinical attachment level, PD=probing depth, WAG=width of attached gingival.

There was statistically significant difference in clinical parameters pre and postoperatively. There was gain in width of attached gingiva from baseline of 0.33±0.47 to 2.92±0.79 mm. There was gain in clinical attachment level from 5.75±1.60 to 2.08±0.98 mm, 9 month postoperatively. There was improvement in probing depth also from 2.08±0.66 to 1.25±0.45 mm. Recession depth correlated positively with root coverage (r=0.89) and inversely with recession width (r=-0.49). 

## DISCUSSION

Results of this study suggested free gingival graft to be a successful procedure both in terms of root coverage and aesthetics. Gingival recession is an issue which is faced both by the clinician and the patient. Various treatment modalities are possible and which procedure is to be chosen depends upon local anatomic conditions, choice of operator and patient’s comfort. The presence of adequate keratinized gingiva serves as a barrier to physical trauma and future progression of recession. There is no universal consensus on amount of attached gingiva for periodontal health, but it is common opinion that area with less than 2 mm of keratinized gingiva is more prone for recession.^16^

Free gingival graft is a versatile mode of treatment which can be used to cover denuded roots and to increase the width of attached gingiva. It can be used as a single step or two step procedure. The technique proposed by Miller is a one-step procedure or the direct approach,^4^ whereas the one described by Bernimoulin *et al.* involves two surgical steps and is referred to as the indirect approach.^17^ Connective tissue grafts has been reported with complete root coverage in class I and II gingival recessions and is usually considered the gold standard.^18^ Complete root coverage has been defined as soft margin at cementoenamel junction, clinical attachment to root, sulcus depth 2 mm or less, no bleeding on probing.^4 ^

It is demonstrated that free gingival graft has less chances of success and predictability as compared to connective tissue grafts.^19^ There are different reasons for incomplete root coverage like improper classification of marginal tissue recession, inadequate root planing, improper preparation of recipient site, inadequate size of interdental papilla, inadequate graft size and thickness, dehydration of donor tissue, inadequate adaptation of graft to root and remaining periosteal bed, failure to stabilize the graft, excess or prolonged pressure in coaptation of sutured graft, reduction of inflammation prior to graft, trauma to graft during initial healing, excessive smoking.^20^

Previous studies have reported coverage of 40-70% using FGG in class I and II recessions.^21^ Free gingival graft was used in this study because: (i) Shallow palatal vault was observed in the studied population which was not suitable for harvesting the connective tissue graft. (ii) The study population presented here with relatively thin gingiva phenotype. Technique such as laterally placed flap could not be employed as chances of donor tissue recession was there. (iii) In all the selected cases there was insufficient apicocoronal gingiva that can’t be placed coronally.^19^^,^^22^

Only mandibular recession defects are studied because mandibular gingiva is aesthetically less demanding for the patients as compared to maxillary gingiva. Moreover, in literature most of the studies present with the combined results of maxillary and mandibular recession defects.^19^ Due to anatomical challenges treatment outcome of both arches are not comparable.^22^ At present, free gingival graft is lagging behind the connective tissue graft but it still holds an edge as far as simplicity and invasiveness of the procedure is concerned. Compared to other techniques, free gingival graft offers unpredictable results regarding color match between donor tissue and recipient site, but studies regarding coverage of gingival recession with free gingival graft is lacking in Indian scenario where due to high melanin pigmentation better aesthetic results can be achieved with this procedure.^14^

Free gingival graft is reported with main disadvantage of aesthetics, but in contrast to previously published papers; satisfactory aesthetic results have been reported in present study as demonstrated by VAS score. Almost 75% of patients were satisfied with aesthetic results. It could be possible due because of studied population has a high degree of melanin pigmentation as compared to studies reported earlier in different population with different degree of melanin pigmentation. Pigmentation reappeared within a period of 6 months which was responsible for high VAS score. It is also possible that the population studied here was more concerned about root coverage and their aesthetic expectations were less. Results of this study indicated 82% root coverage in class I and II Miller recession with fairly acceptable results in terms of esthetics suggesting free gingival graft as a viable option.

## CONFLICT OF INTEREST

The authors declare no conflict of interest.
